# The role of the ICU liaison nurse services on anxiety in family caregivers of patients after ICU discharge during COVID-19 pandemic: a randomized controlled trial

**DOI:** 10.1186/s12912-022-01034-6

**Published:** 2022-09-10

**Authors:** Khadijeh Mottaghi, Shirin Hasanvand, Fateme Goudarzi, Khadijeh Heidarizadeh, Farzad Ebrahimzadeh

**Affiliations:** 1 Master of Critical Care Nursing, Student Research Committee, Lorestan University of Medical Sciences, Khorramabad, Iran; 2 Social Determinants of Health Research Center, School of Nursing and Midwifery, Lorestan University of Medical Sciences, Khorramabad, Iran; 3 Biostatistics, Nutritional Health Research Center, School of Health and Nutrition, Lorestan University of Medical Sciences, Khorramabad, Iran

**Keywords:** Liaison nurse, Anxiety, Caregivers, Intensive Care Units, Patient Discharge, COVID-19

## Abstract

**Background:**

With the onset of the COVID-19 pandemic and the need to maintain social distancing and changes in wards' structure, families no longer access the routine support they received during the hospitalization of their patients in the ICU. This study aimed to determine the effects of ICU liaison nurse services on the anxiety in patients’ family caregivers after ICU discharge during the COVID-19 pandemic.

**Methods:**

This randomized controlled trial was performed in western Iran from February 2020, to March 2021. Sixty subjects were selected from the family caregivers of the patients transferred from the ICU and were randomly assigned to the control (*n* = 30) and the intervention groups (*n* = 30). The control group received routine transfer care. In the intervention group, liaison nurse services were offered in 4 dimensions: patient support, family support, training, support of the ward’s staff, and *the evaluation of the destination ward*. The participants’ anxiety was measured using the Spielberger State Anxiety Inventory immediately after the patient transfer and 6 h after admission to the general ward. Data analyzed with SPSS V16, descriptive and inferential statistics, including Chi-square test, Mann–Whitney test, Wilcoxon test, and Generalized Linear Model with cumulative logit link function. Results were reported at a 0.05 significance level.

**Results:**

A statistically significant difference was observed in baseline anxiety levels (*P* = 0.035) and age group (*P* < 0.001) between the intervention and control groups. After moderating baseline anxiety levels, the age group, and marital status, the impact of the intervention was significant (X^2^ = 10.273, df = 1, *P* < 0.001), meaning that the intervention could reduce the relative chances of developing higher levels of anxiety by 92.1% (OR: 0.08, 95%CI: 0.017–0.373, *P* < 0.001).

**Conclusions:**

This study confirmed the positive impact of nursing services on reducing anxiety in family caregivers during the COVID-19 pandemic. It is recommended to use these services, especially during the COVID-19 condition, to facilitate the patient transfer, support the patient's family, and reduce the health care gap between the ICU and the ward.

**Supplementary Information:**

The online version contains supplementary material available at 10.1186/s12912-022-01034-6.

## Background

The hospitalization of a family member, especially in stressful wards such as the intensive care unit (ICU) or emergency ward (E.W.), can cause anxiety or increase it in the family [1]. Transfer from ICU can also be as traumatic as hospitalization [2] and affect the patient, family, and staff [3], especially the family, who may experience anxiety before, during, and after the transfer process [4]. A study by Op’t Hoog et al. (2020) indicated the importance of the experiences of patient's relatives after the transfer from ICU to the general ward and yielded 5 themes, including relief, uncertainty, the need to be approved for care provision, sharing expectation and the need for continuing care. The patient’s relatives expressed the need for continued care by requesting the presence of a specialist to guide them through all the steps of the transfer process [5] because, at times, families show more negative reactions to the transfer process than patients do [6].

Particularly with the outbreak of COVID-19, due to social distancing guidelines and changes in the wards' structure, families did not have access to the routine support they received during the ICU hospitalization of one of their relatives. They could not even talk with the health care team [7]. In addition, the relationship between relatives and healthcare workers significantly decreased due to the high workload of the staff [8]. During the current pandemic, the Iranian health system imposed some restrictions on patient visitation. Patients’ relatives were to a great extent prohibited from visiting their patients.

On the other hand, especially at the onset of the pandemic, family caregivers avoided visiting their patients due to their fears and the nature of the disease is being unknown to them. Furthermore, due to the high number of hospitalizations and the increased workload of the health care team, providing support for the families decreased significantly. Therefore, separation and distance between the patient and the family increased anxiety and tension due to the abovementioned rules. While in medical and nursing institutions, guidelines were published on various forms of communication [9]. In Iran, little attention has been paid to this issue. Families’ unresolved stress and persistence can lead to the rejection of the treatment plan, the reduced ability of the family to provide support and care for the patient [10], and transfer this anxiety to the patient [11]. Consequently, in critical illness conditions, controlling patients' and families’ anxiety is important because families’ anxiety can affect patients’ recovery [12].

According to Vincent (2019), patients should be seen as a continuum of related incidents from the onset of the disease and ICU hospitalization to recovery. While deciding to hand over the patient from ICU to the general ward, the most critical aspect of post-ICU care has been reported to be proper communication with non-ICU nurses to manage the patients and their families [13]. ICU nurses have an important role in helping families manage anxiety and increasing their ability to cope with stressful situations [14] because they are primarily responsible for managing the transfer process [15]. Many strategies have been developed to improve the quality of nursing care after the patient’s transfer to the ICU, one of which is liaison nurse services [16]. It has been reported that patient visitation in the general ward by a familiar member of the ICU staff may sometimes be effective in addressing many related concerns [13].

Preparing the patient and family for transition to a new environment and supporting them is one of the main responsibilities of the liaison nurse [17]. On the other hand, by having a complementary role, the liaison nurse increases nurses' knowledge, skills, and self-confidence in general wards in dealing with patients with complex needs [4] and enables them to address patients’ care needs [6]. The study by Chaboyer et al. (2004) showed that the ICU liaison nurse could perform a helpful intervention in improving ICU nurses’ attitude toward discharge planning [18]. Another study reported the effectiveness of liaison nurse services in reducing patient discharge delays, effective discharge planning, and increasing the survival rate of the patients at the risk of rehospitalization [19]. However, the studies have reported contradictory results investigating the impact of liaison nurse services on patients' anxiety and vital signs. In the study of Chaboyer et al. (2007), liaison nurse services had no impact on reducing anxiety in family caregivers [4], while in another study, this type of service was reported to be effective in reducing the ICU discharge delay [20]. Noroozi et al. (2019). reported its effective results in reducing anxiety in the patients transferred from the coronary care unit (CCU). Another study investigating the impact of liaison nurse services on physiological parameters of ICU patients reported the ineffectiveness of liaison nurse services [21, 22].

In general, the evidence shows that little research has been conducted on the liaison nurse, especially in Iran and during the COVID-19 pandemic, showing different results [4, 21–23]. According to a study conducted by the current researcher in Iran, liaison nurse services are emerging as a nascent field. The (primary) aim of this paper is to explore the impact of ICU liaison nurse services on anxiety in family caregivers after ICU discharge during the COVID-19 pandemic.

## Methods

### Study design

This study is a single-center randomized controlled trial consisting of two groups and was conducted to evaluate the impact of ICU liaison nurse services on anxiety in family caregivers after ICU discharge. This study was conducted from February 2020, to March 2021. During this time, three COVID-19 peaks appeared in Iran. The first peak emerged in March 2020, and the second and the third peaks in June and September 2020, respectively.

### Setting and participants

Sixty subjects participated in this study who were the family caregivers of the patients transferred from medical and surgical ICUs to general wards of a major teaching hospital affiliated to Lorestan University of Medical Sciences. This 450-bed hospital has 2 ICU wards with a capacity of 10 and 11 beds, in which each nurse provides care to an average of 3 patients. In general wards of the hospital, the nurse-patient ratio depends on the number of patients, which is an average of 6 to 8 patients per nurse. During the COVID-19 pandemic, this hospital admitted COVID-19 patients. The hospital’s area of service has changed, especially during the first peaks of COVID-19, and some wards, including CCU and E.W., were assigned to COVID-related tasks. However, no changes were made in arranging human resources of ICUs, especially during the first peaks of the disease, and a feeling of fear and uncertainty governed the hospital due to the nature of the disease’s being unknown to the health care team members.

The subjects who met the inclusion criteria were assigned to the intervention and control groups using stratified random sampling and a random number table in a 1:1 ratio. The strata included the reason for admission (poisoning and medical-surgical condition), gender (female, male), and the level of education (Below diploma and diploma and above). Inside each stratum, Permuted Block Randomization was implemented, where code A was assigned to the numbers 0–4 and code B, to the numbers 5–9. Sampling was done, at first, from the control group and then from the intervention group to avoid sample contamination. During the sampling process in the control group, each family caregiver assigned to the intervention group according to the random number table was ignored. The opposite was done during the sampling process in the intervention group after the sampling was finished in the control group. According to Table 1,Kutner et al. (2004), the final sample size was calculated to be 23 for each group,, considering

$$\frac{\Delta }{\sigma } = \frac{Difference\,between\,means\,of\,two\,groups}{{Std.\,deviation\,of\,response\,in\,all\,groups}} \simeq 1$$

**Table 1:** Determining sample size (Kutner et al. 2004)

Power 1-β = .90																												
$\frac{\Delta }{\delta }=1.0$					$\frac{\Delta }{\delta }=1.25$				$\frac{\Delta }{\delta }=1.5$				$\frac{\Delta }{\delta }=1.75$				$\frac{\Delta }{\delta }=2.0$				$\frac{\Delta }{\delta }=2.5$				$\frac{\Delta }{\delta }=3$			
α					α				α				α				α				α				α			
r	.2	.1	.05	.01	.2	.1	.05	. 01	.2	.1	.05	.01	.2	.1	.05	.01	.2	.1	.05	.01	.2	.1	.05	. 01	.2	.1	.05	. 01
2	14	18	23	32	9	12	15	21	7	9	11	15	5	7	8	12	4	6	7	10	3	4	5	7	3	3	4	6
3	17	22	27	37	11	15	18	24	8	11	13	18	6	8	10	13	5	7	8	11	4	5	6	8	3	4	5	6
4	20	25	30	40	13	16	20	27	9	12	14	19	7	9	11	15	6	7	9	12	4	5	6	8	3	4	5	6
5	21	27	32	43	14	18	21	28	10	13	15	20	8	10	12	15	6	8	9	12	4	5	6	9	3	4	5	7

Number of groups = *r* = 2, alpha = 0.05, and power = 0.9, which increased to 30 subjects for each group taking into account a sample dropout rate of 30% [24]. Of the 122 caregivers who met the inclusion criteria, 62 withdrew from the research. The final sample size was 30 subjects for both intervention and control groups (Diagram 1).

The inclusion criteria for family caregivers consisted of willingness to participate in the study, obtaining a score of above 20 on the Spielberger’s Anxiety Scale, the caregiver’s not being infected by COVID-19, being over 18 years of age, no previous history of ICU hospitalization in family members, no previous care provision for any other patient, no known history of neurological disease, being the primary and direct caregiver, being the first-degree relative of the patient, willingness to be present at the patient's bedside during the first shift after the patient’s admission to the ward, the patient’s hospitalization in the ICU for at least 24 h, and having no background in the field of medicine. The exclusion criteria are caregivers’ unwillingness to continue participation, change in caregivers during the study, transferring the patient to another hospital, the patient’s discharge with personal consent or direct transfer from the ICU to home, the hemodynamic instability of patient during the study, the occurrence of special conditions which the liaison nurse is not able to manage, in case of sudden incidents (the deterioration of the patient’s condition, need for surgery, death) during the study, and the patient’s readmission in the ICU during the study.

The study began after the ethics committee’s approval and presented the permit to the ICU and the general wards of the selected hospital, and the necessary arrangements were made with the matron. The research process started when the patient transfer order was registered in the medical records, until 6 h after the patient was admitted to the destination ward, i.e., the end of the shift. After the patient transfer order was recorded, the questionnaires were provided to the caregivers who met the inclusion criteria to be self-completed or filled out through an interview.

In order to collect data, a sociodemographic questionnaire was used, including age, gender, marital status, level of education, employment status, satisfaction with the economic status, relationship with the patient, the ward, and duration of time after ICU discharge. The State-Trait Anxiety Inventory (STAI) was used to assess anxiety (the expression of emotions under current circumstances). This scale has 20 items on a 4-point Likert scale (1 = rarely to 4 = almost always). The obtained score falls between 20 and 80, where a score of 20 indicates no anxiety, a score of 21 to 31 shows mild anxiety; 32 to 42, moderate to low anxiety; 43 to 53, moderate to high anxiety; and 54 to 64, relatively severe anxiety. A score of 65 to 75 indicates severe anxiety, and 76 to 80, very severe anxiety [25]. The mentioned tool is psychometrically evaluated in Iran with a Cronbach's alpha of 0.85 for the state anxiety in the Farsi version. The instrument's convergent and content validity has also been confirmed [26]. In the previous study of one of the current paper's authors (the second author), the tool's face validity and content validity are examined, and the test–retest reliability coefficient is calculated to be 0.84 for the whole scale [27]. The research tool was completed through interviews or as self-administered questionnaires by the family caregivers on two occasions, once during the registration of the patient transfer order in the patient’s medical records, and at the end of the first shift, after the patient’s admission in the destination ward. The mean time required to complete the questionnaire was 10 min.

### Control group

In the control group, the patient received routine care. The nurse in charge of the patient's admission was responsible for accepting the patient according to the ward’s routines. The patient was merely informed of the transfer to the general ward. After coordinating with the destination ward, the nurse in charge of the handover transferred the patient to the destination ward. During the transfer process, the nurse gave the nurse in charge of the patient's admission a description of the patient's general condition, actions taken in the ICU, and the measures necessary for follow-ups in the destination ward. Other actions were performed in the ward depending on the data provided in the medical record.

### Intervention group

The participants benefited from liaison nurse services in the intervention group and routine care. They received ICU liaison nurse services, including patient support and care, family education and support, training, and support of the ward’s staff, and the evaluation of the destination ward, with the aim of the structured follow-up on the patient. The services of the ICU liaison nurse continued from Saturday to Thursday during the morning and evening shifts, from the time of the patient’s transfer order until the end of the first shift, after the patient’s admission. The ICU nurse, with a minimum of 8 years of working experience in the ICU and 1 year in the general ward, provides services to patients, their families, and general ward nurses, according to Diagram 2.

According to Diagram 2, the provision of liaison nurse services was made in four domains and two stages as follows:

1. **Before transferring the patient to the destination ward**
  The ICU liaison nurse extracted and recorded the demographic and clinical information of the patients through a checklist or questioning the patient. The liaison nurse explained the transfer order, the reason, and its benefits to the patient. After talking with the patient's family, the liaison nurse identified the patient's main family caregiver, introduced himself/herself to the caregiver, and communicated with him/her. The liaison nurse explained the patient's current condition, causes, and benefits of transferring the patient to the family caregiver. The liaison nurse answered any questions and explained the destination ward and the necessary training about the patient's needs and what the patient was waiting for in the ward. In addition to the training, the necessary emotional support was provided to the patient and the family. According to the needs of the patient in transit, including the existence of a suitable suction system, the condition of the bed (presence or absence of wavy mattress) for patients at risk of bedsores, oxygen delivery equipment, etc., and after making the necessary coordination with the destination and safety the patient was transferred from suitable conditions for transfer. In addition, the liaison nurse informed the ward staff about the patient's transfer and condition at least 1 hour before the patient's transfer, and the intensive care unit was informed of the conditions and facilities of the destination ward.
2. **After the transfer of the patient**
  The liaison nurse was still with the patient and the family and continued to support them and resolve their possible problems until 6 hours after the patient was placed in the destination ward. The contact number of the liaison nurse was given to the main caregiver. The number of visits and attendance at the patient's bedside and the provision of necessary training were tailored to the patient's condition and specifically and based on the patient's needs. An important aspect of the nurse liaison service after the patient transfer was the training and support of the staff in the destination ward. The liaison nurse provided the general nurse with sufficient information about the patient's condition, the nature of his illness, the care plan, and the patient's educational needs. The nurse in charge of patient care, along with the nurse, reviews the various systems of the patient's body, such as respiratory, cardiovascular, nervous, gastrointestinal, etc.; and the care provided in the intensive care unit and the patient's current care needs, and discussed the patient's medication and diet. Staff training and support by the liaison nurse varied according to the request of the destination ward nurse and the case. The contact number of the liaison nurse was provided to the ward.
  The data were analyzed using SPSS software V16 (Serial no.5D1M3E6N5C3G1C1L). Descriptive statistics were used, including frequency, percentage, mean, and standard deviation. In order to compare qualitative variables between the two groups, the Chi-square test, and to compare quantitative variables, the Mann–Whitney test, Wilcoxon signed-rank test, and generalized linear model with cumulative logit link function for ordinal dependent variable were used.

## Results

Sixty samples (30 subjects in each of the two groups) participated in the study. Fisher's exact test showed that no significant difference exists between the intervention and control groups in terms of distribution of variables, including gender, level of education, employment status, satisfaction with the economic status, the reason for hospitalization, marital status, relationship with the patient, the ward, and the time spent between ICU discharge and admission to the general ward. Furthermore, the chi-square test showed a statistically significant difference in the distribution of the variable *age group* between the two groups (X^2^ = 8.75, df:3, *P* = 0.03). A comparison of demographic and clinical variables of the family caregivers and the patients between the control and intervention groups is displayed in Table 2.

**Table 2:** Comparison of clinical and demographic variables between the control and intervention group

Variables	Values	Group				Z/X^2b^	*P*-value
		**Control**		**Intervention**			
		Frequency/mean ^a^	Percentage/SD ^a^	Frequency/mean^a^	Percentage/SD ^a^		
**Age group (years)**	< 25	3	10.0	7	23.3	8.75	0.035
	25–34	4	13.3	7	23.3		
	35–44	1	3.3	5	16.7		
	≥ 45	22	73.3	11	36.7		
**Gender**	Female	16	53.3	14	46.7	0.267	0.797
	Male	14	46.7	16	53.3		
**Level of education**	Below diploma	10	33.3	14	46.7	1.11	0.430
	diploma and above	20	66.7	16	53.3		
**Employment status**	Unemployed	16	53.3	15	50.0	0.067	> 0.999
	Employed	14	46.7	15	50.0		
**Satisfaction with economic status**	low	9	30.0	8	26.7	2.31	0.341
	Moderate	19	63.3	16	53.3		
	High	2	6.7	6	20.0		
**The reason for hospitalization**	Poisoning	16	46.7	16	46.7	0.0	0.999
	No poisoning	14	53.3	14	53.3		
**Marital status**	Single	8	26.7	13	43.3	1.83	0.279
	Married	22	73.3	17	56.7		
**The relationship with the patient**	Father	1	3.3	1	3.3	3.63	0.526
	Mother	1	3.3	1	3.3		
	Siblings	5	16.7	11	36.6		
	Spouse	7	23.3	7	23.3		
	Child	16	53.3	10	33.3		
**Ward**	Medical ICU	18	60.0	14	46.7	1.07	0.438
	Surgical ICU	12	40.0	16	53.3		
**The time between ICU discharge and admission to the general ward (hours)**		3.57	1.45	3.73	1.39	56.09	0.57

^a^ For the variable *time between ICU discharge and entry into the general ward*, as a quantitative variable, the combination of mean and standard deviation was used, while for other quantitative variables, frequency and the percentage were considered

^b^ The statistic X^2^ was calculated for the variables *ward*, *age group*, *gender*, *level of education*, *employment status*, *satisfaction with economic status*, *the reason for hospitalization*, *marital status*, *the relationship with the patient*. The statistic Z was used for the variable time between ICU discharge and admission to the general ward

**Fig. 1:**
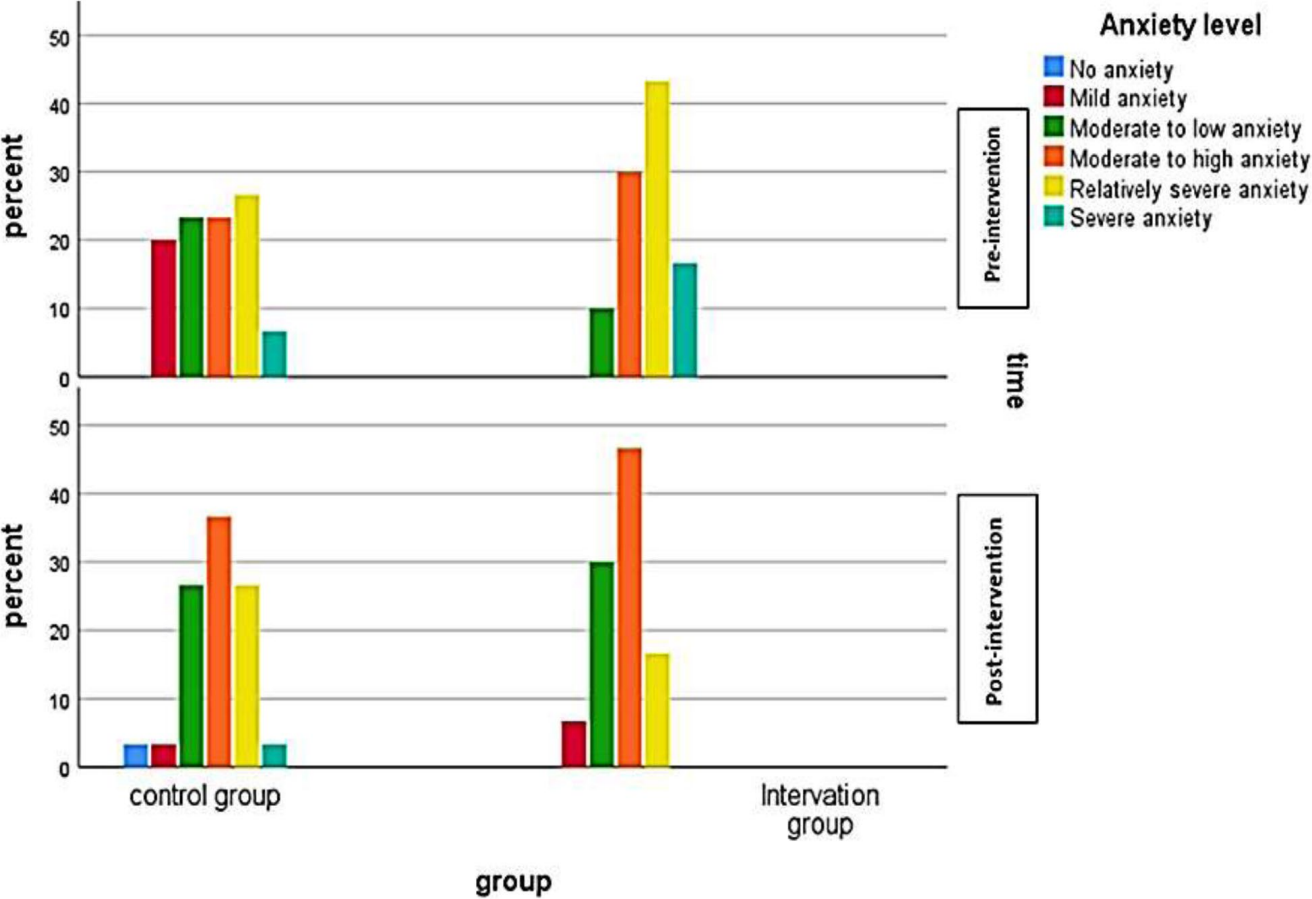
Frequency distribution of the subjects’ levels of anxiety in control and the intervention groups at the beginning and the end of the study

According to Table 3, in the control group, the total relative frequency of the relatively severe and severe anxiety cases was 33.4 at the beginning, which reduced to 30 at the end of the study. While in the intervention group, the total relative frequency of relatively severe and severe cases was from 60 to 16.7 (Table 3). However, results showed a statistically significant difference in the frequency distribution of anxiety levels between the control and the intervention groups (Z = 2.819, *P* = 0.005). At the end of the study, no significant difference in anxiety levels was observed in the control group either at the beginning or end of the study (Z: 0.816, P: 0.415). However, there was a significant difference in anxiety in the intervention group at the study's beginning and end (Z: -4.460, *P* < 0.001). Figure 1 shows the frequency distribution of the subjects’ anxiety levels in the intervention and the control groups on two occasions, the study's beginning and end.

**Table 3:** Frequency distribution of anxiety levels in the control and the intervention groups at the beginning and the end of the study

Levels of the variable anxiety		**Control**		**Intervention**		***P***	**z**
		**Frequency**	**Percent**	**Frequency**	**Percent**		
Pre-intervention anxiety	No anxiety	0	0.0	0	0.0	0.005	2.819
	Mild	6	20.0	0	0.0		
	Moderate to low	7	23.3	3	10.0		
	Moderate to high	7	23.3	9	30		
	Relatively severe	8	26.7	13	43		
	Severe	2	6.7	5	16.7		
Post-intervention anxiety	No anxiety	1	3.3	0	0.0	0.425	0.797
	Mild	1	3.3	2	6.7		
	Moderate to low	8	26.7	9	30		
	Moderate to high	11	36.7	14	46.7		
	Relatively severe	8	26.7	5	16.7		
	severe	1	3.3	0	0.0

The Chi-square test of parallel lines indicated that slope coefficients are the same across response categories $(\chi^{2} = {35}{\text{.960,}}\,{\text{df = 30,}}\,P = 0.209)$. Therefore, the proportional odds assumption was met in the model.

Furthermore, the generalized linear model with cumulative logit link function showed that after moderating baseline values of anxiety, age group, and marital status, the effect of the intervention on the subjects’ level of anxiety was significant (x_2_ = 10.273, df = 1, *p* = 0.001), where the intervention could reduce the odds ratio of any increase in anxiety by 92.1% (adjusted OR:0.079, 95%CI: 0.017–0.373, *P* = 0.001, unadjusted OR:0.069, 95%CI: 0.017–0.279, *P* = 0.001).

## Discussion

The present study was designed to examine the impact of ICU liaison nurse services on family caregivers’ anxiety after ICU discharge during the COVID-19 pandemic. According to the results, ICU liaison nurse services, especially during the COVID-19 pandemic, have effectively reduced family caregivers’ anxiety levels. In line with the present results, the previous studies have demonstrated that the liaison nurse effectively reduces anxiety in ICU patients and their family caregivers.

The study results by Tabanejad et al. (2014) were also consistent with the current ones, showing that using liaison nurse services has increased patient and family satisfaction with the services of liaison nurses [19]. Another study showed that after providing liaison nurse services, the anxiety in the intervention group subjects was significantly reduced, reflecting the impact of the services on patients’ transfer anxiety during the transfer from the coronary ICU [23]. Another study also reported decreased patients' anxiety after receiving the desired services [21]. Using the liaison nurse effectively reduces anxiety in the caregivers of the patients discharged from ICUs. In a qualitative study by Op’t Hoog et al. (2020), families explicitly expressed the need for a liaison nurse during the transfer process [5]. According to Brooke et al. (2012), providing information to understand the destination environment can significantly reduce patients’ and families' transfer anxiety compared to the standard care procedures [28]. In a study conducted by Li et al. (2022), The liaison nurse service during the COVID-19 pandemic showed that after the intervention, the total family satisfaction score of ICU patients in the intervention group, satisfaction with the care and information provided was significantly higher than the control group. The level of relocation anxiety in patients in the intervention group was significantly lower than the control group after the intervention [29]. This study was not performed during the COVID-19 peak, unlike the present study. In a study by Keen et al. (2022), participants' descriptions show that the implementation of liaison nurse services during the COVID-19 pandemic reduced the nurse's moral distress and developed close family relationships [30].

During the outbreak of COVID-19, the health staff was unable to communicate effectively with families due to clinical burdens, which led to families’ dissatisfaction [7]. Such shortages increase families’ uncertain and stressful experiences [30]. The impact of liaison nurses was really important in the early days of the COVID-19 pandemic when the visitation rules were very strict, and family members could only visit their patients in severe cases. This relationship enables family members to support their loved ones and maintain safety during hospital stays [30]. Communication has been identified as a critical component in the range of liaison nurses’ activities. Poor communication, care coordination, and information exchange between the health care professionals of ICU and the ward increase the risks of poor-quality discharge and may lead to severe complications, ICU rehospitalization, and increased mortality rate [31]. Poor interactions were considered as the major source of staff’s stress. Insufficient data on the case for answering the family's questions and poor communication between the physician and the family increases nurses’ stress [32]. In this study, efforts were made to address communications issues to a large extent by creating coordination between the ICU and destination wards and providing sufficient explanations to the family about the transfer handover process, all in the form of liaison nurse services. During the COVID-19 pandemic, Gabbie et al. (2020) developed a family liaison team to improve the patient's relationship with the family in intensive care units; their studies suggest that intervention is adequate [7]. In the Netherlands, Klop et al. (2021) formed a family support team and contacted the relatives of ICU patients via phone. The relatives’ experience of the intervention was positive [8]. Lipworth et al. (2020) developed a telecommunication program to support the ICU ward and patients’ families, the result showing high levels of satisfaction among liaison nurses who informally expressed positive feedback [33]. Similar to the present study, this study was performed simultaneously with the coronavirus infection wave and the occupation of a large proportion of intensive care beds.

Furthermore, in a combined study, Lopez-Soto et al. (2021) investigated the effect of the family liaison team on relatives and friends’ relationship with therapists [9], where the mentioned method was considered an easy approach resulting in relatives’ high levels of satisfaction. A mentioned study was performed during the first surge of the COVID 19 pandemic in a tertiary London hospital.

Although all these studies were in line with the current study results, they referred to telecommunication as the major solution. However, in the present study, due to the suspension of patient visitation and the possibility of one family member at the patient's bedside, training and support were provided to family caregivers, mainly in person. In some instances, these services were offered remotely via phone.

However, this finding contradicts previous studies, which suggested that liaison nurse services did not impact some outcomes concerning patients and caregivers. In the study by Chaboyer et al., no difference was observed in patients' and caregivers’ anxiety between the control and the intervention groups [4]. This result may be explained because Chaboyer et al. did not have a baseline criterion for measuring the participants’ anxiety. Furthermore, in an Iranian clinical trial study, a trained liaison nurse followed up on the intervention group subjects for two weeks. However, no significant difference was observed in the level of anxiety between the control and the intervention groups immediately either after discharge or one week and two weeks afterward [34]. This discrepancy could be attributed to the ambiguities in the definition of the liaison nurse’s role. This study, ignorant of the broad scope of the liaison nurse’s role, defines the most important role of the liaison nurse merely as someone who trains patients and their families after discharge and the scope of the liaison nurse's activities was not well defined.

Another possible explanation for this inconsistency is that the time of primary assessment of anxiety was not similar for all the participants in this study. The patients discharged from the hospital who were transferred to the ward were not divided into separate groups, impacting the subjects’ anxiety. In the present study, liaison nurse services did not solely focus on the patient, and efforts were made to consider the factors affecting family caregivers’ level of anxiety. In the current study, the services mentioned above were commenced prior to patient transfer and continued until admission in the destination ward.

One of the most striking results emerging from the data is that liaison nurse services were able to reduce the relative chances of any higher levels of anxiety by 92.1%, which can be associated with the use of liaison nurse services over a longer period, for at least one shift after admission in the general ward, which is longer than some studies. It can also be due to providing support for caregivers despite the pandemic and the high workload of health care team members. The transfer experience depends on preparing the patient and the family for the first contact with the health care team in the ward and the time of transfer and cooperation and making plans to allocate sufficient time for transfer, especially in complicated cases [35]. Families can provide the necessary psychological and social support to their patients only if they can understand the transfer process and are assured of the safety of the general ward [28]. The patients transferred from the ICU to general wards are readmitted to the ICU prior to discharge due to inadequate care services and hemodynamic instability in the ward [36]. Receiving information from the health care team is very important for the family caregivers of ICU survivors, according to the experimental studies conducted on their experiences and needs [37]. In the present study, this issue was addressed based on the related topics in the liaison nurse services.

Furthermore, evidence shows that showing empathy and offering information and support to the relatives of ICU patients are important factors during their stay in this unit [5]. The stress in the family members of ICU patients may increase during a global pandemic and strict visitation limitations [30]. They are exposed to many of these stressors, and the stress also increases due to COVID-related restrictions for family and friends in visiting the patient [38]. In Iran, no appropriate support system exists for the patients discharged from the ICU and their family caregivers even under normal circumstances, much less during the COVID-19 pandemic, which puts extra stress on both patients and their families and health care providers.

According to the nurses, the hospital managers have no correct understanding of the condition in general wards and the patients transferred from the ICU. As a result, the organization's structure and leadership significantly impact nurses’ ability to provide the required care to patients transferred from the ICU [39]. Liaison nurse services lead to facilitated transfer, better preparation of the medical staff for receiving the patient, increased clinical competency of the ward’s staff in providing care for patients, and reducing family caregivers’ anxiety [6], which in turn reduces the ICU hospitalization rate and the length of hospital stay and significantly decreases mortality after ICU discharge [40]. In addition, continuing care reduces discharge delays, improves understanding of the exchanged information, and prevents adverse incidents [31].

One of the strengths of this study is the duration of the liaison nurse's service during the patient care transition. So that the mentioned services for one shift continued after the patient was placed in the general ward. Also, the target people for receiving nursing services was not just patients or their families. General ward nurses also benefited from these services. One of the limitations of this study is not taking the destination ward into account as a measurable variable. Another limitation was the absence of samples from the night shift, affecting the results.

## Conclusion

In the present study, given the services provided by the liaison nurse, a significant decrease was observed in family caregivers’ anxiety. Family and patients are looking for a simple way to enhance communication and address patients’ needs. Considering the advantages of the liaison nurse and its role in facilitating the transfer and reducing the anxiety of family caregivers, and also given the fact that using these services is a cost-effective solution to decrease the possible complications of transfer, it is recommended to implement these services, especially during COVID-19 pandemic, in order to facilitate the transfer, support the family, and reduce the care gap between the ICU and the ward. Furthermore, future research should investigate the experiences of family caregivers, patients, and health care team members in qualitative studies.

## Supplementary Information

**Additional file 1: Diagram 1.** Flow chart of the study.

**Additional file 2: Diagram 2.** A description of liaison nurse services.

## Data Availability

The datasets generated and/or analyzed during the current study are not publicly available due to confidentiality but are available from the corresponding author on reasonable request.

## Abbreviations

ICU: Intensive Care Unit
E.W.: Emergency Ward
STAI: State-Trait Anxiety Inventory
CCU: Coronary Care Unit
