# Supplementary material for: The role of the ICU liaison nurse services on anxiety in family caregivers of patients after ICU discharge during COVID-19 pandemic: a randomized controlled trial
Source: BMC Nurs. 2022 Sep 10;21:253. doi: 10.1186/s12912-022-01034-6 (PMC9464053; doi:10.1186/s12912-022-01034-6)
Supplement: Supplementary file 1 — Additional file 1: Diagram 1. Flow chart of the study. [file 12912_2022_1034_MOESM1_ESM.docx]

**Diagram 1. Flow chart of the study**

Analysed (n= 30 )
♦ Excluded from analysis (give reasons)(n=0 )

Analysed (n=30 )
♦ Excluded from analysis (give reasons)(n=0 )

Lost to follow-up (give reasons) (n=0)

Discontinued intervention (give reasons) (no subject)

Lost to follow-up (give reasons) (n=0)

Discontinued intervention (give reasons) (no subject)

Randomized (n= 120)

Assessed for eligibility (n=122)

Excluded (n= 62)

**.** Not being present in the retest session: 2

**.** Allocated to the intervention group while performing sampling in the control group: 30

**.** Allocated to the control group while performing sampling in the intervention group: 30

## Follow-Up

## Analysis

## Enrollment

Allocated to intervention (n= 60)

♦ Received allocated intervention (n=30 )

♦ Did not receive allocated intervention (give reasons) (n= 30 )

## Allocation

Allocated to intervention (n=60)

♦ Received allocated intervention (n=30 )

♦ Did not receive allocated intervention (give reasons) (n= 30)
