# Supplementary material for: The role of the ICU liaison nurse services on anxiety in family caregivers of patients after ICU discharge during COVID-19 pandemic: a randomized controlled trial
Source: BMC Nurs. 2022 Sep 10;21:253. doi: 10.1186/s12912-022-01034-6 (PMC9464053; doi:10.1186/s12912-022-01034-6)
Supplement: Supplementary file 2 — Additional file 2: Diagram 2. A description of liaison nurse services. [file 12912_2022_1034_MOESM2_ESM.docx]

Diagram 2 . A description of liaison nurse services
